# Quality of Life in Endometrial Cancer Survivors: What Does Obesity Have to Do with It?

**DOI:** 10.1155/2011/308609

**Published:** 2011-10-29

**Authors:** Amanda Nickles Fader, Heidi E. Frasure, Karen M. Gil, Nathan A. Berger, Vivian E. von Gruenigen

**Affiliations:** ^1^Division of Gynecologic Oncology, Greater Baltimore Medical Center/Johns Hopkins Hospital, Baltimore, MD 21204, USA; ^2^Department of Obstetrics and Gynecology, University Hospitals Case Medical Center, Cleveland, OH 44106, USA; ^3^Department of Obstetrics and Gynecology, Akron General Medical Center, Akron, OH 44302, USA; ^4^Center for Science, Health and Society, Case Western Reserve University School of Medicine, Cleveland, OH 44106, USA; ^5^Department of Obstetrics and Gynecology, Summa Akron City Hospital, Akron, OH 44309, USA

## Abstract

*Objective*. Most women with type I endometrial cancer (EC) are obese, increasing the risk of morbidity and mortality in this population. The study objective was to evaluate the impact of obesity on quality of life (QOL) and general health status in EC survivors with early-stage disease. *Methods*. A prospective ancillary analysis of stage I/II EC survivors. The association of BMI with QOL questionnaire variables measured with the functional assessment of cancer therapy (subscales: physical (PWB), functional (FWB), social, and emotional well-being) and the physical (PCS) and mental component summary subscales of the short-form medical outcomes survey was determined. *Results*. 152 women completed both questionnaires; 81% were obese. After multiple linear regression, BMI was inversely associated with PWB (*P* = .001), FWB (*P* = 0.048), and PCS (*P* = .001). *Conclusions*. Despite the good prognosis associated with early-stage EC, QOL, and physical health are not optimized in obese survivors. This paper highlights the importance of incorporating health-related QOL assessments and obesity interventions during the survivorship period.

## 1. Introduction

Outcomes research in the last decade has evolved beyond investigating conventional clinical endpoints to incorporating quality of life (QOL) endpoints, or direct assessments of how patients feel [1]. To that end, both the NCI and FDA have mandated that the goals of cancer therapies should be to improve both survival and QOL [1, 2]. As a result of earlier detection and improvement in therapies, many women with a cancer diagnosis are living longer, and therefore, there has been considerable interest in studying health-related QOL outcomes in cancer survivors after treatment. 

Women with endometrial cancer comprise a large segment of female cancer survivors. Endometrial cancer, the most common gynecologic malignancy in the USA, was diagnosed in over 42.000 women in 2010 [3]. One of the most significant risk factors for this cancer is obesity. Most women with endometrial cancer are overweight or obese and possess significant obesity-driven comorbidities that threaten their long-term health and QOL [4, 5]. It is well established that obese women without cancer have poorer health outcomes and QOL than their nonobese counterparts [6]. However, little is known about the effects of obesity on QOL and physical health of endometrial cancer survivors beyond the completion of therapy.

 Several epidemiologic studies have linked obesity with an increased rate of death from all causes, including cancer [4, 7–12]. A recent prospective cohort study examining the relationship of obesity to mortality in males and females diagnosed with several different cancers found that the relative risk of cancer-related death in morbidly obese women with endometrial cancer was 6.25—the highest rate of all deaths reported amongst the study subjects [11]. It is evident from this and other reports that although most endometrial cancer survivors have a good cancer prognosis with the potential for long-term survival, their obesity puts them at risk for morbidity and mortality. Despite this, most obese endometrial cancer survivors do not adopt weight loss or healthier lifestyle modifications after their cancer diagnosis [12]. 

We hypothesized that increasing obesity would adversely impact general health and QOL in this cancer patient cohort. The aims of this study were to investigate the effect of obesity on general health status and QOL in women with early-stage endometrial cancer. 

## 2. Materials and Methods

### 2.1. Patients

After obtaining informed consent and institutional review board approval, prospective ancillary QOL data were obtained from three previously published trials on women with stage I-II endometrial cancer [5, 12, 13]. These trials were conducted at two participating gynecologic oncology centers in Northeastern Ohio (University Hospitals Case Medical Center, Cleveland, Ohio, and Akron General Medical Center, Akron, Ohio). Of note, although two of these studies were interventional trials, all QOL assessments were made prior to any interventions. Women who underwent a primary surgical staging procedure with or without adjuvant radiation therapy and were recurrence-free at the time of their post-treatment interview were eligible. QOL questionnaires were completed a minimum of 6 months and a maximum of 36 months after primary surgery/adjuvant treatment. 

Demographic data were collected by interview with a research assistant and from medical record chart review. Height and weight were obtained at the time of questionnaire completion by a nurse. Body mass index (BMI) was calculated (weight (kg) divided by height (m^2^)) and categorized as normal/overweight (18.5–29.9), obese (30–39.9), or morbidly obese (≥40).

### 2.2. Instruments

General health status was measured with the short-form medical outcomes survey (SF-36), a comprehensive survey designed to measure physical and mental health. Scales on this questionnaire include the physical component summary (PCS) and mental component summary (MCS) [14]. This 36-question scale assesses eight health concepts: (1) limitations in physical activities because of health problems; (2) limitations in social activities because of physical or emotional problems; (3) limitations in usual role activities because of physical health problems; (4) bodily pain; (5) general mental health (psychological distress and well-being); (6) limitations in usual role activities because of emotional problems; (7) vitality (energy and fatigue); (8) general health perceptions. Fatigue was also specifically measured (as defined by ≤46 on the SF-36 vitality subscale) [15]. 

QOL was measured with the functional assessment of cancer therapy (FACT-G), a 27-item core questionnaire evaluating physical, functional, social, and emotional well-being within the previous 7 days (PWB, FWB, SWB, and EWB). The FACT-G is a reliable and validated instrument for measuring QOL in cancer patients, including the elderly [16, 17].

### 2.3. Statistical Analysis

One-way comparisons of variance (ANOVA) with posthoc analysis was used to compare FACT-G domains and SF-36 measures between categories of BMI (normal/overweight (BMI ≤ 30), obese (BMI 30.0–39.9), and morbidly obese (BMI ≥ 40). Items within each domain are summed, with higher scores indicating higher QOL. BMI was analyzed as a continuous variable, and Spearman correlation coefficients were calculated for BMI with SF-36 PCS, MCS, and FACT-G domains. In addition, Spearman correlation coefficients were calculated for individual line items of the four FACT-G domains and for the eight SF-36 subscales with BMI. Individual items within the FACT-G were left as worded for the correlation analysis. 

Multiple regression analysis was used to assess the effect of BMI on FACT-G domain and SF-36 summary scores while controlling for patient demographic, clinical, and treatment (radiation therapy) variables. Variables included were age, educational level, marital status, radiation treatment, time since diagnosis, and smoking. Educational level was categorized as <16 years versus ≥16 years (college degree or higher), and smoking was categorized according to <5.0 versus ≥5.0 pack years. Continuous variables in the model included age, BMI, and time since diagnosis (months). Variables were regressed on individual FACT-G domains (PWB, FWB, SWB, and EWB) and SF-36 summary measures (PCS, MCS) using a stepwise linear regression procedure. Variables were entered in the regression models at a probability level of *P* < 0.05 and were removed at a probability level >0.10. Collinearity diagnostics (tolerance and variance inflation factor) were examined to assess multicollinearity of variables included in the final model. Exploratory analysis using the SF-36 vitality subscale was conducted to assess fatigue in this dataset. Presence of fatigue was defined as ≤46 on the vitality SF-36 subscale [18]. SPSS version 14.0 (Chicago, Ill) was used for analysis.

## 3. Results

 One hundred fifty-two women completed questionnaires within 6–36 months after treatment (median 14.6 months). Demographic data is presented in Table 1. Mean subject age was 57, and performance status was zero in 95%. Forty two percent of subjects graduated from college, 61% were married, 93% percent had stage I disease, and less than half of the women received adjuvant radiation treatment. Eighty-one percent of the women were obese (mean BMI was 37.5 kg/m^2^). Forty-five percent of participants completed the questionnaire within 1 year of their cancer diagnosis, 20% within 2 years, and 35% at 2-3 years from diagnosis. Notably, the timing of questionnaire administration was not significantly different among the normal/overweight versus obese and morbidly obese patients (*P* = .210), nor did QOL scores significantly differ based on questionnaire timing. 

One-way ANOVA revealed a significant difference overall among BMI groups for the SF-36 PCS score and the PWB domain FACT-G (Table 2). On post hoc analysis, morbidly obese endometrial cancer patients had a significantly lower SF-36 PCS score than their normal/overweight (*P* = 0.002) and obese counterparts (*P* = .05). For FACT-G PWB, obese and morbidly obese patients had a significantly lower score as compared to normal/overweight patients (*P* = .004 and *P* = .001, resp.). Scores did not differ between obese and morbidly obese patients (*P* = .800) for the PWB domain. 

To further assess the impact of increasing BMI on QOL measures and general health, correlations were performed with FACT-G domains and SF-36 summary measures (Table 3). BMI was inversely correlated with PWB and FWB domain scores. Within the PWB domain, BMI was correlated with several line items, including “lack of energy” and “meeting needs of family.” Within the FWB domain, “ability and fulfillment of work,” “enjoyment of life,” “things I usually do for fun,” and “content with QOL right now” were all inversely correlated with BMI. On SF-36, BMI was inversely correlated with PCS total score and all subscales except for mental health. 

Multiple regression analyses were conducted to examine the impact of BMI on FACT-G and SF-36 scores while controlling for patient demographic and clinical variables (Table 4). BMI remained a significant factor affecting PWB and FWB domains of the FACT-G (*P* = .001, *P* = 0.048) and SF-36 PCS (*P* < .001). Of note, age was a significant factor affecting SF-36 PCS and MCS scores, and the FACT-G EWB score and marital status also significantly impacted FACT-G PCS, with unmarried women having significantly poorer scores. The combination of increasing age and BMI further decreased QOL scores. (standardized beta for BMI = −.420, age = −.367). Smoking was also a significant factor affecting FACT-G PWB and EWB. 

Fatigue was present in 43 (28.3%) of women. Mean (SD) BMI of those having fatigue was 41.0 (8.7) as compared to 36.5 (8.6) without fatigue (*P* = .006). Finally, 61 patients (40%) received adjuvant radiation therapy; treatment did not impact FACT-G or SF-36 scores or presence of fatigue.

## 4. Discussion

Previous research has demonstrated that obesity in women without cancer is associated with poorer health-related quality of life [18, 19]. A recent study of 9094 female and male participants in the Canadian Multicentre Osteoporosis Study demonstrated that increasing BMI, particularly in women, had a significant negative impact on health and QOL [7]. Similarly, our study demonstrates that increasing obesity is associated with lower health-related QOL in endometrial cancer survivors, as demonstrated in analysis of several domains of the FACT-G and SF-36 scales. On domain line-item analysis, BMI was not only significantly correlated with poor physical health scores and lack of energy but also inversely correlated with several functional domain scores including lack of fulfillment of work and enjoyment of life. Because endometrial cancer is often diagnosed at an early stage, and therefore, may be curable, enhancing the quality of life of survivors is a high priority. Yet QOL gains are elusive in this population as there is a high prevalence of obesity among survivors, and therefore, a significant proportion of survivors with associated sedentary lifestyles and possession of obesity-driven comorbidities. Physical inactivity and obesity affect survivors' health-related QOL, either independently or through their association with chronic diseases. These and other obesity-driven factors put endometrial cancer survivors at increased risk for major public health problems such as heart disease, diabetes, osteoarthritis, and stroke. Furthermore, according to a prospective observational cohort study by Calle et al., obese survivors are also at risk of recurrent endometrial cancer and other malignancies as well when compared to their thinner counterparts [11]. 

However, few studies have addressed the impact of obesity and its associated comorbidities on QOL in this cancer cohort. Limitations of prior research have included heterogeneous gynecologic populations and different adjuvant therapies [20–22]. Klee and Machin assessed 49 women with endometrial cancer who received adjuvant radiation therapy and concluded that most patients experience physical side effects and have overall lower QOL compared to a matched population of healthy women [20]. van de Poll-Franse noted good overall QOL in endometrial cancer survivors although QOL scores were lower in those who had received adjuvant therapy [22]. By contrast, in our study, poor overall physical domain and health-related QOL scores were observed. This may be due to the more obese population represented in our analysis when compared to the European studies referenced above. Furthermore, our study did not note any difference in scores between women who had received adjuvant radiation versus those who did not. This may be because subjects were surveyed several months after the completion of therapy, and therefore, QOL differences based on adjuvant treatment status may have not been apparent. 

It is understood that increasing BMI and a sedentary lifestyle has an adverse effect on health and QOL on cancer survivors. In a study of 386 endometrial cancer survivors who were surveyed about exercise and BMI, investigators showed that lack of exercise and excess body weight exacerbated treatment-related declines in QOL [6]. Multivariate analyses showed that healthy, fit endometrial cancer survivors reported significantly better QOL than did unfit, obese survivors and that both exercise (*P* < 0.001) and BMI (*P* < 0.001) were independently associated with QOL. While obesity is a leading risk factor for endometrial cancer and also increases the risk of cancer-related and all-cause mortality in survivors, previous work from our group demonstrates that the majority of survivors are not making lifestyle modifications that positively impact upon their obesity or associated comorbidities after cancer treatment [5, 12]. When surveyed, most female cancer survivors want to make positive lifestyle modifications, with the most common goals being improving physical activity, performing meaningful activities, losing weight, and eating a better diet [23]. von Gruenigen et al. demonstrated that only 12% of endometrial cancer survivors exhibited lifestyle behaviors recommended by the American Cancer Society for cancer survivors, including adequate physical activity, five servings of fruit and vegetables per day, and no smoking [12]. Basen-Engquist et al. also reported in a separate QOL survey study of 121 endometrial cancer survivors that most women exhibited a sedentary lifestyle which significantly impacted their health-related QOL and, as a general matter, were not making positive lifestyle modifications after a diagnosis of cancer [24]. 

Apart from the impact BMI had on perceived physical health and functioning in the current study, we also demonstrated that morbid obesity in cancer survivors was significantly associated with fatigue. Although we did not employ a specific fatigue instrument to study this variable, previous studies have demonstrated that the use of the SF-36 vitality subscale is a reasonable surrogate tool for measuring fatigue [18, 25, 26]. Fatigue is a common side effect of cancer and its treatment, and it frequently goes unrecognized and untreated [26]. While the etiology of fatigue is unclear, numerous clinical factors, such as obesity, are likely contributors. It is also quite possible that fatigue may prevent these cancer survivors from achieving healthier lifestyles through dietary modification and exercise. Therefore, these issues should be addressed with all endometrial cancer patients in the immediate posttreatment period when patients are most likely to be motivated to modify their lifestyles. 

Multiple linear regression analyses confirmed the adverse influence of higher BMI as well as increased age and smoking on health-related QOL. Groessl et al. demonstrated in 1326 elderly adults (mean age 72) from the Rancho Bernardo longitudinal cohort study that obese older adults had lower QOL than those who are overweight or of normal weight [19]. This supports the findings in our study that BMI has a significant impact on physical health and QOL independent of age. Our study demonstrated that the additive effects of obesity and age in elderly endometrial cancer survivors further decreased QOL scores. Physicians caring for endometrial cancer survivors must, therefore, have an understanding of the relationship between BMI, obesity-driven comorbidities, and post cancer-treatment symptom stress as well as knowledge of patients' functional/psychologic status and potential age-related limitations that may impact on their physical and mental health. 

Study strengths include the prospective design and the use of both general and specific validated QOL instruments. This report is one of the most comprehensives examining the impact of obesity on health-related QOL for endometrial cancer survivors. Our study is limited by the post hoc analysis, lack of racial diversity, possible selection bias, lack of standardized data on physical activity, and by variations in the timing of posttreatment interviews. However, 45% of surveys were completed within the first year after cancer diagnosis, and timing of the surveys did not impact QOL scores significantly. In an effort to address these limitations and comprehensively address the question of obesity and quality of life in endometrial cancer survivors, our research group is actively conducting a prospective lifestyle interventional trial. 

## 5. Conclusions

Improving QOL should be a priority in the management of cancer, especially for long-term survivors. Obesity poses one of the greatest health threats to early-stage, endometrial cancer patients after treatment and adversely impacts their QOL. Morbidly obese endometrial cancer survivors have an increased risk of low summary and line-item scores for several health-related QOL domains, signifying the importance of considering such factors in programs aimed at obesity intervention for this population. These patients require a comprehensive approach to medical care, and interventional trials focused on weight reduction are needed to assess the feasibility of improving specific QOL domains, general health status, and long-term outcomes in this survivor population. Furthermore, trials of endometrial cancer treatment should include a health-related QOL assessment as this measure may lead to improvement of clinical care by emphasizing the patients' perspectives on the health and QOL benefits of cancer therapy.

## Figures and Tables

**Table 1:** Patient demographics and clinical variables, *n* = 152.

Age, mean (SD), range	57.7 (11.0), range 32–87
Performance status	
0	145 (95%)
1	7 (5%)
Body mass index (kg/m^2^), mean (SD), range	37.5 (8.7), range 22.5–64.7
<30	29 (19.1%)
30–39	69 (45.4%)
≥40	54 (35.5%)
Adjuvant radiation therapy	61 (40%)
Smoking	
Never	111 (73.0%)
<5.0 pack years	5 (3.3%)
≥5.0 pack years	36 (23.7%)
Education	
High school	45 (29.6%)
Some college	42 (27.6%)
College graduate or higher	65 (42.8%)
Stage of cancer	
I	141 (92.8%)
II	11 (7.2%)
Married	93 (61.2%)
Selected comorbidities	
Hypertension	70 (46.1%)
Diabetes mellitus	34 (22.4%)
Time since diagnosis (months), median	14.6
Race	
Caucasian	143 (94.1%)
African American	7 (4.6%)
Other	2 (1.3%)

*n* (%) presented unless otherwise noted.

**Table 2:** FACT-G and SF-36 means overall and by body mass index.

	Normal/overweight (*n* = 29)	Obese (*n* = 69)	Morbidly obese (*n* = 54)	*P* value*
SF-36				
PCS	49.7 (9.7)	45.7 (9.2)	41.4 (10.9)	.001
MCS	54.8 (9.0)	50.7 (10.2)	50.8 (12.1)	.217
FACT-G				
PWB	26.5 (1.4)	23.9 (4.0)	23.5 (3.4)	.001
SWB	19.2 (4.6)	18.1 (4.5)	17.5 (4.1)	.293
EWB	20.0 (3.6)	19.8 (4.0)	19.1 (4.8)	.632
FWB	22.5 (6.3)	21.8 (5.4)	21.0 (5.2)	.492

Total	88.1 (11.5)	83.6 (13.5)	81.2 (14.1)	.098

Mean (SD) values presented.

*one-way ANOVA for comparison of normal/overweight, obese, and morbidly obese groups.

**Table 3:** Correlations between FACT domains, SF36 (PCS, MCS), and BMI.

FACT Domain	Body mass index
Physical	−.320**
Lack of energy	.363**
Nausea	.114
Meeting needs of family	.264**
Pain	.125
Side effects of treatment	.001
Feel ill	.115
Forced to spend time in bed	.108
Functional	−.175*
Ability to work	−.187*
Fulfillment of work	−.201*
Able to enjoy life	−.208*
Accepted my illness	−.132
Sleeping well	.073
Enjoy things I usually do for fun	−.228**
Content with QOL right now	−.216**
Social	−.142
Closeness to friends	.170*
Emotional support from family	.011
Support from friends	−.013
Family acceptance of illness	−.024
Satisfied with family communication	.144
Close to partner	−.015
Satisfied with sex life	−.095
Emotional	−.081
Sadness	.116
Satisfied with coping with illness	−.016
Losing hope	.137
Nervous	.037
Worry about dying	.065
Worry condition will get worse	.041
PCS	−.326**
Physical functioning	−.389**
Role physical	−.220**
Bodily pain	−.189*
General health	−.325**
MCS	−.090
Vitality	−.256**
Social functioning	−.231**
Role emotional	−.213**
Mental health	−.094

*P < .05.

**P < .01.

**Table 4:** Multiple linear regression analysis for each FACT-G domain and SF-36 PCS and MCS scores.

	Variables	Standardized Beta coefficient	*t* value	*R*, adjusted *R*2	*F* value, *P* value
FACT-G					
PWB	BMI	−.218	−2.72	.329, .089	5.69, *P* = .001
	married	−.206	−2.56		
	smoking	−.168	−2.11		
SWB	time since diagnosis	−.296	−3.71	.296,.081	13.73, *P* < .001
EWB	smoking	−.218	−2.70	.273,.061	5.71, *P* = .004
	age	.169	2.10		
FWB	BMI	−.165	−2.00	.165, .020	3.99, *P* = .048
SF-36					
PCS	BMI	−.420	−5.44	.468	20.24, *P* < .001
	age	−.367	−4.76	.209	
MCS	age	.207	2.55	.207, .036	6.51, *P* = .012
